# Reversible Rings with Involutions and Some Minimalities

**DOI:** 10.1155/2013/650702

**Published:** 2013-12-30

**Authors:** W. M. Fakieh, S. K. Nauman

**Affiliations:** Faculty of Science, King Abdulaziz University, P.O. Box 80203, Jeddah 21589, Saudi Arabia

## Abstract

In continuation of the recent developments on extended reversibilities on rings, we initiate here a study on reversible rings with involutions, or, in short, ∗-reversible rings. These rings are symmetric, reversible, reflexive, and semicommutative. In this note we will study some properties and examples of ∗-reversible rings. It is proved here that the polynomial rings of ∗-reversible rings may not be ∗-reversible. A criterion for rings which cannot adhere to any involution is developed and it is observed that a minimal noninvolutary ring is of order 4 and that a minimal noncommutative ∗-reversible ring is of order 16.

## 1. Introduction

Throughout this note we assume that rings are associative may be without identity. We will specifically mention if a ring is with the identity. In most of the cases the rings are equipped with an involution that we refer to by ∗.

A ring *R* is termed as a reversible ring by Cohn in [1] if for any pair of elements *x*, *y* ∈ *R*, *xy* = 0, then *yx* = 0. Anderson and Camillo used the notation *ZC*_2_ for the same type of rings in [2]. Recently, the notion of reversibility is extended to *α*-reversibility in [3] and strong *α*-reversibility in [4], where *α* : *R* → *R* is an endomorphism. Thus, if *x*,  *y* ∈ *R*, such that *xy* = 0⇒*yα*(*x*) = 0, then *α* is termed as right reversible in [3] and if the converse holds in the sense that *xα*(*y*) = 0⇒*yx* = 0, then *α* is termed as right strong *α*-reversible in [4]. The ring *R* is called right *α*-reversible and right strong *α*-reversible, respectively. Analogously, the terms *α*-reversible and strong *α*-reversible are defined.

In this note we replace the endomorphism *α* by an involution ∗ which is an anti-automorphism on *R* of order two. Thus, the *α*-reversibility is replaced by ∗-reversibility that will be defined in Section 2. Note that neither *α*-reversibility is ∗-reversibility nor ∗-reversibility is *α*-reversibility, because clearly, in general, an anti-automorphism cannot be an endomorphism, and, conversely, an endomorphism cannot be an anti-automorphism. Though some results of these notions may go parallel, we will work on ∗-reversibility from scratch.

A ring *R* is zero ring if *R* ^2^ = 0 and a domain if *R* is a no-zero-divisors ring (see [5]), that is, without non-zero zero divisors. *R* is called reduced if *R* has no non-zero nilpotent elements and symmetric if for any triple *a*_1_, *a*_2_, *a*_3_ ∈ *R*, *a*_1_ *a*_2_ *a*_3_ = 0, then *a*_*s*(1)_ *a*_*s*(2)_ *a*_*s*(3)_ = 0, where *s* : {1,2, 3}↦{1,2, 3} is a permutation. Symmetric rings were introduced by Lambek in [6]. In [2], the notation *ZC*_3_ is used for a symmetric ring. It is known that every reduced ring is symmetric [2, 6] and neither a symmetric ring is reversible nor a reversible ring is symmetric (for this debate and examples and counterexamples, see [2, 7–9]). For a ring with identity, it is clear that every symmetric ring is reversible. All these types of rings are semicommutative, where a ring *R* is semicommutative, in the sense of Bell [10], if for any pair of elements *x*, *y* ∈ *R*, *xy* = 0⇒*xry* = 0, for all *r* ∈ *R*. Semicommutative rings have many names in the literature. For details and examples and counter examples, see [11]. A ring *R* is reflexive if for any pair of elements *x*, *y* ∈ *R*, *xRy* = 0 then *yRx* = 0. Symmetric and reversible rings are reflexive.

In Section 2, we have given some properties and several examples and counter examples related to ∗-reversible rings. In Section 3, we have modified an example [12, Example 2] to verify that the polynomial rings of ∗-reversible rings may not be ∗-reversible.  Section 4 deals with identifying minimal left or right symmetric, symmetric, reflexive, reversible, and ∗-reversible rings. In particular, a criterion is developed for rings which are noninvolutary; that is, a ring which cannot adhere to involutions.

For elementary notions about rings we refer to [13, 14] and for rings with involution to [15–18].

## 2. ∗-Reversible Rings

**Definition 1 s**

Let *R* be a ring with the involution ∗. We say that an element *x* ∈ *R* is right ∗-reversible if there is a non-zero element*y* ∈ *R*, such that *xy* = 0, then yx* = 0. Similarly, let us call an element *x* ∈ *R* right ∗-inverse-reversible if there is a non-zero element *y* ∈ *R*, such that xy* = 0, then *yx* = 0. Analogously, we define left ∗-reversible, ∗-reversible, left ∗-inverse-reversible, and ∗-inverse-reversible elements.

If all elements of the ring *R* are right (left, two-sided) ∗-reversible or ∗-inverse-reversible, then we use the same term for the ring *R*.

**Proposition**

For any ring *R* with the involution ∗, the following are equivalent:

(1) *R*  is right ∗-reversible,

(2) *R* is left ∗-reversible,

(3) *R* is right ∗-inverse-reversible,

(4) *R* is left ∗-inverse-reversible.

**Proof**

(1)⇒(2) Let for any pair of non-zero elements *x*, *y* ∈ *R* and *x* and *y* annihilate each other in the direction *xy* = 0, which implies by definition that yx* = 0 and then again x*y* = 0. Hence, finally, y*x** = y*x = 0. The rest can be proved analogously.

In the light of the above proposition if all elements of a ring which annihilate each other are left (or right) ∗-reversible (or left or right ∗-inverse-reversible), then they are ∗-reversible. Hence, we have the following.

**Definition**

If *R* satisfies any one of the conditions of Proposition 2, then we say that *R* is a reversible ring with the involution ∗ or that *R* is a ∗-reversible ring.

**Example 4**

(1) All domains with some involution ∗ are ∗-reversible. For instance, the commutative ring $R=\mathbb{Z}[\sqrt{-5}]=\{x+\sqrt{-5}y:x,y\in \mathbb{Z}\}$ is reversible with the involution ∗ defined by ${\left(x+\sqrt{-5}y\right)}^{*}=x-\sqrt{-5}y$. The ring of real quaternions *ℍ* is reversible with the natural involution ∗ defined on its elements by (*a* + *bi* + *cj* + *dk*)* = *a* − *bi* − *cj* − *dk*.

(2) Among the nondomains, if, for some involution ∗, a domain *D* is ∗-reversible, then the cartesian product *D* × *D* under the induced involuton is ∗-reversible, where the induced involution on *D* × *D* is defined by (*d*_1_, *d*_2_)* = (*d*_1_*, *d*_2_*), for all *d*_1_, *d*_2_ ∈ *D*.

Consider the product *R* = *ℤ*_10_ ⊕ *ℤ*_10_ which is a commutative ring under usual multiplication. Let ∗ be an involution on *R* defined by (*a*, *b*)* = (*b*, *a*), for all (*a*, *b*) ∈ *R*. Now, if we let *x* = (6,0), *y* = (5,2); then we see that *xy* = 0 while y*x = (2,5)(6,0) = (2,0) ≠ 0. Hence, *R* is not ∗-reversible. Clearly, *R* is reduced. Hence, one concludes that a ring with some involution ∗ may be commutative and reduced but not ∗-reversible.

Note that *ℤ*_4_ is not reduced but *ℤ*_4_ is reversible with the trivial involution.

(3) For any ring *R* the ring of strictly upper triangular matrices SUTM_3_(*R*) (or strictly lower triangular matrices SLTM_3_(*R*)) is not ∗-reversible for any involution ∗ on *R* (see details in Example 23).

(4) An involution ∗ on a ring *R* is an anisotropic involution if there exists no *x* ∈ *R*∖0, such that xx* = 0; otherwise it is called isotropic [17, 19]. Let us say that a ring *R* is anisotropic (isotropic) if it adheres to an anisotropic (isotropic) involution.

For example, the rings $\mathbb{Z}[\sqrt{-5}]$ and *ℍ* in Example 4(1) are anisotropic.

On the other hand, the noncommutative quaternion algebra *Q* (see [17, page 25]) over any field *𝔽* with char(*𝔽*) ≠ 2 and with a basis {1, *i*, *j*, *k*},

(1) Q:={x=a+bi+cj+dk:  a,b,c,d,∈𝔽,i2,j2∈𝔽×,  ij=k=−ji},

and with a natural involution defined by x* = *a* − *bi* − *cj* − *dk* may not be ∗-reversible. For instance, if *𝔽* = *𝔽*_3_, and *i* ^2^ = *j* ^2^ = −1 then (1 + *i* + *j*)(1 − *i* − *j*) = 0 but (1 + *i* + *j*)^2^ ≠ 0.

The group ring *ℤ*_2_(*Q*_8_) (where *Q*_8_ is the group of real quaternions), as discussed in [8, Example 7], is reversible and is not symmetric. Let us define an involution on its elements

(2) x=a1+a2x−1+a3xi+a4x−i+a5xj+a6x−j +a7xk+a8x−k, ∀ai∈ℤ2

by

(3) x∗=a1+a2x−1+a3x−i+a4xi+a5x−j+a6xj +a7x−k+a8xk, ∀ai∈ℤ2.

Then xx* = 0 if and only if ∑_*i*=1_^8^ *a*_*i*_^2^ = 0. This holds even though *x* ≠ 0. For instance, if *x* = 1 + *x*_*i*_ + *x*_*j*_ + *x*_*k*_, then one calculates that xx* = 0. Hence, *ℤ*_2_(*Q*_8_) is not ∗-reversible and it is isotropic under ∗.

(5) See [19, Example 2]. Consider the group ring *ℂ*[*S*_3_], where *S*_3_ = {1, *σ*, *σ* ^2^, *τ*, *τσ*, *τσ* ^2^} is the symmetric group on three letters. *ℂ*[*S*_3_] adheres to an involution ∗ defined by (∑_*g*∈*S*_3__ *r*_*g*_ *g*)* = (∑_*g*∈*S*_3__ *r*_*g*_ *g* ^−1^). Assume that *α* = (1/6)(∑_*g*∈*S*_3__ *g*), *β* = (1/6)(1 + *σ* + *σ* ^2^ − *τ* − *τσ* − *τσ* ^2^), and *γ* = (1/3)(2 − *σ* − *σ* ^2^). Then *ℂ*[*S*_3_]*α* and *ℂ*[*S*_3_]*β* are anisotropic and ∗-reversible while *ℂ*[*S*_3_]*γ* is isotropic and not ∗-reversible, so the ring *ℂ*[*S*_3_] = *ℂ*[*S*_3_]*α* ⊕ *ℂ*[*S*_3_]*β* ⊕ *ℂ*[*S*_3_]*γ* is isotropic and not ∗-reversible.

(6)  *Examples of left or right *∗*-reversible elements of a ring.* In Proposition 2, though it is determined that a one-sided ∗-reversible or ∗-inverse-reversible ring is just ∗-reversible, this criterion does not hold for individual elements. For example, consider the ring of matrices *M*_*n*_(*R*) over any ring *R*, with or without 1. Then *M*_*n*_(*R*) is an involution ring with the involution ∗, where, if *A* ∈ *M*_*n*_(*R*), then A* ∈ *M*_*n*_(*R*) is the transpose of *A*. If A* = *A*, then *A* is a symmetric matrix.

Now, let *A*, *B* ∈ *M*_*n*_(*R*), such that *B* is symmetric and that *AB* = 0. Then (*AB*)* = BA* = 0. Hence, *A* is right ∗-reversible.

Note that, a right (or left) ∗-reversible element may not be a left (or right) ∗-reversible. For instance, in case of elementary matrices in *M*_2_(*ℤ*), *e*_11_ *e*_21_ = 0⇒*e*_12_ *e*_11_ = 0, but *e*_21_ *e*_11_ = *e*_21_ ≠ 0. So *M*_2_(*ℤ*) is not ∗-reversible, but it is anisotropic, because, for all *A* ∈ *M*_2_(*ℤ*), AA* = 0⇒*A* = 0.

A criterion for the equality of different ∗-reversible elements is the following.

**Proposition**

For any reversible ring *R* with the involution ∗ and for any element *x* ∈ *R*, the following are equivalent:

(1) *x* is right ∗-reversible,

(2) *x* is left ∗-reversible,

(3) *x* is right ∗-inverse-reversible,

(4) *x* is left ∗-inverse-reversible.

**Proof**

Proofs can be obtained directly by Definitions 1.

By the above proposition we conclude that if *R* is reversible, then any left or right ∗-reversible or ∗-inverse-reversible element is simply termed as a ∗-reversible element.

If a ring is ∗-reversible, then we have the following.

**Proposition**

Every ∗-reversible ring is (1) symmetric, (2) reversible, (3) reflexive, and (4) semicommutative.

**Proof**

(1) Let *R* be a ∗-reversible ring. Assume that for any *x*, *y*, *z* ∈ *R*, *xy* *z* = 0, then yzx* = 0 or zx*y* = 0 or that x*y*z* = 0. Using the double involutions we get *z* *yx* = 0.

Again, *xy* *z* = 0 means that z*y*x* = 0 or that (y*x*)*z* = (*xy*)z* = 0 implies *z* *xy* = 0.

Using *z* *xy* = 0, by the similar arguments as above, *yx* *z* = 0. Finally, by symmetry we get *yzx* = 0 and *xzy* = 0. Hence, *R* is symmetric.

(2) It is proved in the first line of the proof of Proposition 2.

(3) and (4) These are obvious.

For any subset *X* of a ring *R*, the right and left annihilators of *X* in *R* are denoted by *r*_*R*_(*X*) and *l*_*R*_(*X*), respectively. In particular, if *X* = {*x*}, then we use the terminologies *r*_*R*_(*x*) and *l*_*R*_(*x*).

Let *R* be an involution ring with the involution ∗. Because a ∗-reversible ring *R* is semicommutative, so every left or right annihilator of *R* is an ideal (see [20, Lemma 1.1]). We restate here Proposition 2.3 of [4] in terms of ∗-reversible rings which provides some additional information. The proof of each equivality is elementary.

**Proposition**

Let *R* be an involution ring with an involution ∗. Then the following are equivalent:

(1)  *R* is a ∗-reversible ring;

(2)   *r*_*R*_(x*) = *r*_*R*_(*x*) for each element *x* ∈ *R*;

(3)   *l*_*R*_(x*) = *l*_*R*_(*x*) for each element *x* ∈ *R*;

(4)   *l*_*R*_(x*) = *r*_*R*_(*x*) for each element *x* ∈ *R*;

(5)   *r*_*R*_(x*) = *l*_*R*_(*x*) for each element *x* ∈ *R*;

(6)–(9) replace x* and *x* by subsets S* and *S* of *R*, respectively, in (2)–(5);

(10) for any two non-empty subsets *A* and *B* of *R*, *AB* = 0 if and only if BA* = 0 if and only if B*A = 0.

An element *a* ∈ *R* is called symmetric with respect to ∗ if a* = *a*.

**Proposition**

Let *R* be a ring with 1 and with an involution ∗.

(1) If *R* is ∗-reversible, then every idempotent in *R* is symmetric with respect to ∗. In particular, 1* = 1.

(2) Let *e* be a central idempotent. Then *eR* and (1 − *e*)*R* are ∗-reversible if and only if *R* is ∗-reversible.

(3) *R* is ∗-reversible if and only if, for every central idempotent *e* ∈ *R*, *eR* is ∗-reversible.

(4) Let *R* be abelian (i.e., every idempotent of *R* is central). Then *R* is ∗-reversible if and only if, for every idempotent *e* ∈ *R*, *eR* is ∗-reversible.

**Proof**

(1) Indeed, if *e* ^2^ = *e*, then *e*(1 − *e*) = 0 implies that (1 − *e*)e* = 0, or e* = ee* = *e*. Hence, in particular, 1* = 1.

(2) Let *R* be ∗-reversible. Let *e* ∈ *R* be an idempotent. Then by (1)  e* = *e*, and so *eR* and (1 − *e*)*R* are ∗-subrings of *R*. Hence, these are ∗-reversible.

Conversely, let *eR* and (1 − *e*)*R* be ∗-reversible. Let *xy* = 0 for some *x*, *y* ∈ *R*. Then *exey* = 0. So, by hypothesis, eyex* = *e* yx* = 0.

Again by hypothesis,

(4) $$\begin{array}{c} \left(1-e\right)x\left(1-e\right)y=\left(1-e\right)xy=0, \end{array}$$

which implies that

(5) $$\begin{array}{c} \left(1-e\right)y\left(1-e\right)x^{*}=\left(1-e\right)yx^{*}=0. \end{array}$$

Hence, we conclude that yx* = *e* yx* = 0.

(3) and (4) follow from (2).

**Proposition**

Let *R* and *S* be rings and let *t* : *R* → *S* be an isomorphism. Then we have the following.

(1) ∗**- **is an involution on *R* if and only if   *t*(∗): = *t*∘∗∘*t* ^−1^ is an involution on *S*.

(2) An element *x* ∈ *R* is right (left, two-sided) ∗-reversible if and only if its image *t*(*x*) ∈ *S* is right (left, two-sided) *t*(∗)-reversible.

(3) An element *x* ∈ *R* is right (left, two-sided) ∗-inverse-reversible if and only if its image *t*(*x*) ∈ *S* is right (left, two-sided) *t*(∗)-inverse-reversible.

(4) *R* is ∗-reversible if and only if *S* is *t*(∗)-reversible.

(5) *R* is ∗-inverse-reversible if and only if *S* is *t*(∗)-inverse-reversible.

**Proof**

(1) Clearly, *t*∘∗∘*t* ^−1^ : = *t*(∗) is an anti-automorphism on *S* of order two and conversely *t* ^−1^∘*t*(∗)∘*t* = ∗ is an anti-automorphism on *R* of order two. It is a routine work to check that if ∗ is an involution on *R* then *t*(∗) is an involution on *S* and conversely if *t*(∗) is an involution on *S* then ∗ is an involution on *R*.

(2) Let 0 ≠ *x* ∈ *R* be right ∗-reversible and for some 0 ≠ *y* ∈ *R*, *xy* = 0. Then yx* = 0. The image of *x* in *S* is *t*(*x*), and, naturally, the image of x* in *S* is *t*(*x*)^*t*(∗)^. Thus, *t*(*xy*) = *t*(*x*)*t*(*y*) = 0 in which both products are non-zero and this implies that

(6) $$\begin{array}{c} t\left(yx^{*}\right)=t\left(y\right)t\left(x^{*}\right)=t\left(y\right)t{\left(x\right)}^{t(*)}=0. \end{array}$$

Hence, *t*(*x*) ∈ *S* is right *t*(∗)-reversible.

Conversely, if 0 ≠ *x*′ ∈ *S* is *t*(∗)-reversible, then there is 0 ≠ *y*′ ∈ *S*, such that *x*′*y*′ = 0 which gives *y*′*x* ^′*t*(∗)^ = 0. But there exist 0 ≠ *x* ∈ *R* and 0 ≠ *y* ∈ *R* such that *t* ^−1^(*x*′) = *x* and *t* ^−1^(*y*′) = *y*. So *t*(*x*) = *x*′ and

(7) $$\begin{array}{c} t\left(x^{*}\right)=t{\left(x\right)}^{t(*)}=x^{\prime t(*)}\Rightarrow t^{-1}\left({x^{\prime }}^{t(*)}\right)=x^{*}. \end{array}$$

This means that

(8) $$\begin{array}{c} t^{-1}\left(x^{\prime }y^{\prime }\right)=t^{-1}\left(x^{\prime }\right)t^{-1}\left(y^{\prime }\right)=xy=0, \end{array}$$

which implies that

(9) $$\begin{array}{c} t^{-1}\left(y^{\prime }x^{\prime t(*)}\right)=t^{-1}\left(y^{\prime }\right)t^{-1}\left(x^{\prime t(*)}\right)=yx^{*}=0. \end{array}$$

Hence, *x* ∈ *R* is right ∗-reversible.

The proofs of the remaining parts of (2) and that of (3),(4), and (5) are analogous.

Central idempotents play important role in a direct sum decomposition of a ring. If *R* is a ring with involution and *R*_1_ is a direct summand of *R*, then *R*_1_ can be given a structure of an involution ring [15, Lemma 2.3] and the converse also holds naturally. Then it follows from Propositions 8 and 9 and from Section 7 of [13] the following.

**Theorem 10**

All direct sums and direct summands of ∗-reversible rings are ∗-reversible.

**Example 11 s (trivial extensions)**

Let *R* be any ring; a trivial extension *T*(*R*, *R*) of *R* is a subring of the upper triangular matrix ring over *R* and is defined as

(10) $$\begin{array}{c} T\left(R,R\right)=\left\{\left[\begin{array}{cc} r & s\\ 0 & r \end{array}\right]:r,\;\;s\in R\right\}. \end{array}$$

Define an involution ∗ on the ring

(11) $$\begin{array}{c} T\left({\mathbb{Z}}_{p},{\mathbb{Z}}_{p}\right)=\left\{\left[\begin{array}{cc} x & y\\ 0 & x \end{array}\right]:x,\;\;y\in {\mathbb{Z}}_{p}\right\}, \end{array}$$

where *p* is a prime, by

(12) $$\begin{array}{c} {\left[\begin{array}{cc} x & y\\ 0 & x \end{array}\right]}^{*}=\left[\begin{array}{cc} x & -y\\ 0 & x \end{array}\right]. \end{array}$$

Clearly, *T* is ∗-reversible.

Note that for any prime *p*, *ℤ*_*p*_ is reduced. If we replace *p* by a composite number such that *ℤ*_*p*_ is not reduced, then *T* may not be ∗-reversible. For instance, one can check that *T*(*ℤ*_4_, *ℤ*_4_) is not ∗-reversible; however, *T*(*ℤ*_6_, *ℤ*_6_) is ∗-reversible. We further have the following.

**Theorem**

If *R* has an involution ∗, then the involution *T*(∗) on *T*(*R*, *R*) defined by

(13) $$\begin{array}{c} {\left[\begin{array}{cc} r & s\\ 0 & r \end{array}\right]}^{T(*)}=\left[\begin{array}{cc} r^{*} & s^{*}\\ 0 & r^{*} \end{array}\right],\;\forall \left[\begin{array}{cc} r & s\\ 0 & r \end{array}\right]\in T\left(R,R\right) \end{array}$$

is an involution. If *R* is reduced and ∗-reversible, then the trivial extension *T*(*R*, *R*) is *T*(∗)-reversible.

**Proof**

It is a routine work to check that *T*(∗) is an involution on *T*(*R*, *R*).

Assume that *R* is reduced and ∗-reversible. Let

(14) $$\begin{array}{c} \left[\begin{array}{cc} r & s\\ 0 & r \end{array}\right]\left[\begin{array}{cc} r^{\prime } & s^{\prime }\\ 0 & r^{\prime } \end{array}\right]=0. \end{array}$$

Then

(15) $$\begin{array}{c} rr^{\prime }=0=rs^{\prime }+sr^{\prime }. \end{array}$$

By the ∗-reversible property, *r*′r* = 0. Moreover, because every reduced ring is symmetric and semicommutative, so by (15) *srr*′ = *rsr*′ = 0 which implies that *r* ^2^ *s*′ = 0. Then (*rs*′)^2^ = 0 which gives *rs*′ = 0 or that *s*′r* = 0. Again by (15) and *rs*′ = 0, we get *sr*′ = 0; hence, *r*′s* = 0. Combining these we conclude that

(16) [r′s′0r′][rs0r]T(∗)=[r′s′0r′][r∗s∗0r∗]=[r′r∗r′s∗+s′r∗0r′r∗]=0.

*The Dorroh Extension*. Let *A* be an algebra over a commutative ring *C*. The Dorroh extension of *A* by *C* is a ring *D* = *A* × *C* in which sum of elements is defined componentwise and the product is defined by the rule

(17) $$\begin{array}{c} \left(a_{1},c_{1}\right)\left(a_{2},c_{2}\right)=\left(a_{1}a_{2}+a_{1}c_{2}+a_{2}c_{1},c_{1}c_{2}\right). \end{array}$$

If the algebra *A* adheres to an involution ∗, then an induced involution ∗_*D*_ on *D* is (*a*, *c*)^∗_*D*_^ : = (a*, *c*) for every (*a*, *c*) ∈ *D*. We prove the following.

**Theorem**

Let *C*, be an integral domain and *A* an algebra with 1 over *C*. Then *A* with an involution ∗ is ∗-reversible if and only if its Dorroh extension *D* is ∗_*D*_-reversible.

**Proof**

Let *A*, *C* and *D* be as given in the hypothesis with *A* as ∗-reversible. Consider two non-zero elements (*a*_1_, *c*_1_), (*a*_2_, *c*_2_) of *D* such that (*a*_1_, *c*_1_)(*a*_2_, *c*_2_) = 0. Then we prove that (*a*_2_, *c*_2_)(*a*_1_, *c*_1_)^∗_*D*_^ = 0. Clearly, *c*_1_ *c*_2_ = 0 implies that either *c*_1_ = 0 or *c*_2_ = 0. Assume first that *c*_2_ = 0. Then

(18) $$\begin{array}{c} a_{1}a_{2}+a_{2}c_{1}=\left(a_{1}+1\cdot c_{1}\right)a_{2}=0 \end{array}$$

and so

(19) $$\begin{array}{c} a_{2}{\left(a_{1}+1\cdot c_{1}\right)}^{*}=0=a_{2}a_{1}^{*}+a_{2}c_{1}, \end{array}$$

where 1* = 1 (see Proposition 8(1)) and (*ac*)* = a*c (by the definition of an involution on an algebra over a ring). Thus, we get

(20) $$\begin{array}{c} a_{2}a_{1}^{*}+a_{1}^{*}c_{2}+a_{2}c_{1}=0 \end{array}$$

which means that

(21) $$\begin{array}{c} \left(a_{2},c_{2}\right){\left(a_{1},c_{1}\right)}^{{*}_{D}}=0. \end{array}$$

same conclusion can be obtained if we take *c*_1_ = 0. The converse is obvious.

## 3. The Polynomial Rings of ∗-Reversible Rings

Note that if a ring is commutative, reduced, or Armendariz, then so is *R*[*x*]. But if *R* is semicommutative, reversible, or symmetric, then *R*[*x*] may not be either semicommutative, reversible, or symmetric. These were established in [12, Example 2], [20, Example 2.1], and [21, Example 3.1], respectively, by providing the same counterexample. We continue this example to prove that if *R* is ∗-reversible with some involution ∗, then *R*[*x*] may not be ∗-reversible. To get the goal we will make some modification in the example.

**Example**

A noncommutative ring with (or without) identity is symmetric and reversible but not ∗-reversible with some involution ∗.

Let *a*_0_, *a*_1_, *a*_2_, *b*_0_, *b*_1_, *b*_2_, and *c* be some mutually noncommutative indeterminates and let *T* = *ℤ*_2_[*a*_0_, *a*_1_, *a*_2_, *b*_0_, *b*_1_, *b*_2_, *c*] be a free polynomial algebra. Consider the ring *ℤ*_2_ + *T*, and define an ideal *I* generated by the expressions

(22) a0b0,a0b1+a1b0,a0b2+a1b1+a2b0,a1b2  +a2b1,a2b2,a0rb0,a2rb2,b0a0,b0a1+b1a0,b0a2+b1a1+b2a0,b1a2  +b2a1,b2a2,b0ra0,b2ra2,(a0+a1+a2)r(b0+b1+b2),  (b0+b1+b2)r(a0+a1+a2),r1r2r3r4,

where *r*, *r*_1_, *r*_2_, *r*_3_, *r*_4_ ∈ *T*. The factor ring *R*_1_ = (*ℤ*_2_ + *T*)/*I* is reversible [20, Example  2.1] and symmetric [21, Example  3.1].

Define an involution on *ℤ*_2_ + *T* by 0* = 0, 1* = 1, *a*_i_* = *b*_*i*_, and *b*_i_* = *a*_*i*_; for all *i* = 0,1, 2 and c* = *c*. This is a routine work to check that this is an involution on *ℤ*_2_ + *T* and that I*⊆*I* and obviously *T* ^4^⊆*I*. Define the induced involution on *R*_1_ by setting ${\left(\bar{x}\right)}^{*}=\bar{x^{*}}$, for all $\bar{x}\in R_{1}$. Now $\bar{a_{0}}\bar{b_{0}}=\bar{0}$, but $\bar{b_{0}}{\bar{a_{0}}}^{*}=\bar{b_{0}}\bar{b_{0}}\neq \bar{0}$. Hence, *R*_1_ is not ∗-reversible.

For the without identity part, one may notice that, in *ℤ*_2_ + *T*, *ℤ*_2_ seems to be a superfluous part just to bring the identity in the system. *I* is still an ideal of *T* and the ring *R*_1_′ = *T*/*I* satisfies all claims as stated in [20, Example 2.1] and [12, Example 3.1]. The involution *T* can be defined by setting *a*_i_* = *b*_*i*_, *b*_i_* = *a*_*i*_; for  all  *i* = 0,1, 2 and c* = *c*, and the induced involution on *R*_1_′ can be obtained as previously done. With this involution *R*_1_′ is not ∗-reversible.

Note that (*ℤ*_2_ + *T*)[*x*] or just *T*[*x*] are domains, so these are symmetric, reversible, and ∗-reversible, but the factor rings *R*_1_[*x*] and *R*_1_′[*x*] are not.

**Example 15**

A noncommutative ring is symmetric, reversible, and ∗-reversible for some involution ∗.

We make some modification in Example 14. Let the ring *T* be as in Example 14, and let the ideal *J* be generated by the set

(23) {a02,b02,a22,b22,a0b0,b0a0,a2b2,b2a2,a0ra0,b0rb0,a0rb0,a2rb2,b0ra0,b2ra2,a2ra2,b2rb2,b0a1+b1a0,a0b1+a1b0,a1b2+a2b1,a0a1+a1a0,a1a2+a2a1,b0b1+b1b0,b1b2+b2b1,b1a2+b2a1,a0a2+a12+a2a0,a0b2+a1b1+a2b0,b0a2+b1a1+b2a0,b0b2+b12+b2b0,(b0+b1+b2)r(a0+a1+a2),(a0+a1+a2)r(b0+b1+b2),(a0+a1+a2)r(a0+a1+a2),(b0+b1+b2)r(b0+b1+b2),r1r2r3r4},

where *r*, *r*_1_, *r*_2_, *r*_3_, *r*_4_ ∈ *T*.

**Theorem**

(i) The factor ring *R*_2_′ = *T*/*J*  (or *R*_2_ = (*ℤ*_2_ + *T*)/*J*) is (15) reversible, (2) symmetric, (3) semicommutative, and (4) ∗-reversible for some involution ∗.

(ii)  *R*_2_′[*x*]  (or *R*_2_[*x*]) does not satisfy any of (1), (2), (3), or (4).

**Proof**

If (4) holds, then (1), (2) and (3), by default, followed from Proposition 6. We only prove (4) for *R*_2_′; such a proof for *R*_2_ follows automatically.

First define the involution on *T* and the induced involution on *R*_2_′ as in Example 14. Clearly, *T* ^4^⊆*J* and J*⊆*J*.

So, for (4), assume that ∗ is an involution on *R*_2_′ which is induced from *T*. As previously stated, in [12, 20, 21], let us call each product of the indeterminates *a*_0_, *a*_1_, *a*_2_, *b*_0_, *b*_1_, *b*_2_, *c* a monomial and say that *f* ∈ *T* is a monomial of degree *n* if it is a product of exactly *n* number of these indeterminates. Let *H*_*n*_ be the set of all linear combinations of such monomials of degree *n*.  *H*_*n*_ is finite and the ideal *J* is homogeneous in the sense that if ∑_*i*=1_^*k*^ *r*_*i*_ ∈ *J*, with *r*_*i*_ ∈ *H*_*i*_, then every *r*_*i*_ ∈ *J*.

We will prove first thatif *f*_1_ *g*_1_ ∈ *J* with *f*_1_, *g*_1_ ∈ *H*_1_, then *g*_1_ *f*_1_* ∈ *J*. The different cases for this situation are as under

(24) $$\begin{array}{c} \left(f_{1}=a_{0},g_{1}=a_{0}\right),\;\;\left(f_{1}=b_{0},g_{1}=b_{0}\right),\\ \left(f_{1}=b_{2},g_{1}=b_{2}\right),\;\;\left(f_{1}=a_{2},g_{1}=a_{2}\right),\\ \left(f_{1}=a_{0},g_{1}=b_{0}\right),\;\;\left(f_{1}=a_{2},g_{1}=b_{2}\right),\\ \left(f_{1}=b_{0},g_{1}=a_{0}\right),\;\;\left(f_{1}=b_{2},g_{1}=a_{2}\right),\\ \left(f_{1}=a_{0}+a_{1}+a_{2},g_{1}=b_{0}+b_{1}+b_{2}\right),\\ \left(f_{1}=b_{0}+b_{1}+b_{2},g_{1}=a_{0}+a_{1}+a_{2}\right). \end{array}$$

First four are identical; so, if *a*_0_ *a*_0_ ∈ *J*, then we see that *a*_0_ *a*_0_* = *a*_0_ *b*_0_ ∈ *J*. The same holds for the remaining.

Second four are also identical: so if *a*_0_ *b*_0_ ∈ *J*, then we see that *b*_0_ *a*_0_* = *b*_0_ *b*_0_ ∈ *J*. Same holds for the remaining.

For the second last we have:

(25) f1g1=a0b0+(a0b1+a1b0)+(a0b2+a1b1+a2b0) +(a1b2+a2b1)+a2b2∈J;

then,

(26) g1f1∗=b0b0+(b0b1+b1b0)+(b0b2+b1b1+b2b0) +(b1b2+b2b1)+b2b2∈J,

and the same holds for the last one.

Now let *f*, *g* ∈ *T*, such that *fg* ∈ *J*. Then we will prove that gf* ∈ *J*. Assume that *f* = ∑_*i*=1_^*k*^ *f*_*i*_ and *g* = ∑_*j*=1_^*l*^ *g*_*j*_. Then *fg* = *f*_1_ *g*_1_ + *f*_1_ *g*_2_ + *f*_2_ *g*_1_ + *k*, where all monomial in *k* are of degree 4, so *k* ∈ *J*. By the above *f*_1_ *g*_1_ ∈ *J*, so *f*_1_ *g*_2_ + *f*_2_ *g*_1_ ∈ *J*. Then

(27) $$\begin{array}{c} gf^{*}=g_{1}f_{1}^{*}+g_{1}f_{2}^{*}+g_{2}f_{1}^{*}+k^{*}. \end{array}$$

Again, from the above *g*_1_ *f*_1_* ∈ *J* and as k* ∈ *J*, we only need to prove that *g*_1_ *f*_2_* + *g*_2_ *f*_1_* ∈ *J*. With the option *f*_1_ = *a*_0_, *g*_1_ = *a*_0_, let us pick *f*_2_ = *r* ∈ *J* and *g*_2_ = *b*_0_ *t* where *t* is some monomial. Then

(28) $$\begin{array}{c} g_{1}f_{2}^{*}+g_{2}f_{1}^{*}=a_{0}r^{*}+b_{0}ta_{0}^{*}=a_{0}r^{*}+b_{0}tb_{0}\in J. \end{array}$$

For the option *f*_1_ = *b*_0_ + *b*_1_ + *b*_2_, *g*_1_ = *a*_0_ + *a*_1_ + *a*_2_, let us pick *g*_2_ = *r* ∈ *J* and *f*_2_ = *s*(*a*_0_ + *a*_1_ + *a*_2_). Then

(29) g1f2∗+g2f1∗=(a0+a1+a2)(s(a0+a1+a2))∗ +r(b0+b1+b2)∗=(a0+a1+a2)(a0+a1+a2)s∗ +r(a0+a1+a2)∈J.

Similarly, the remaining options and the possible combinations such that *fg* ∈ *J* can be simplified to prove that gf* ∈ *J*. Hence, we conclude that *R*_2_′ is ∗-reversible.

(ii) The common argument which everyone poses is the following. Let *f*(*x*) = *a*_0_ + *a*_1_ *x* + *a*_2_ *x* ^2^, *g*(*x*) = *b*_0_ + *b*_1_ *x* + *b*_2_ *x* ^2^ ∈ *R*_2_′[*x*]. Then *f*(*x*)*g*(*x*) ∈ *J* but *f*(*x*)*cg*(*x*) ∉ *J*. So, *R*_2_′[*x*] is not semicommutative. Hence, it is neither reversible nor symmetric. There is no question of ∗-reversibility as well. The same holds for *R*_2_[*x*].

A ring *R* is Armendariz, as introduced in [22], if, for any commuting indeterminate *x*, the polynomials *f*(*x*), *g*(*x*) ∈ *R*[*x*] are such that *fg* = 0, and if *a*_*i*_ ∈ Coef(*f*) and *b*_*j*_ ∈ Coef(*g*), then *a*_*i*_ *b*_*j*_ = 0. For instance, if *R* is reduced, then *R* is Armendariz. A favourable conditional case is the following.

**Theorem 17**

If *R* is an Armendariz ring, then *R* is ∗-reversible if and only if *R*[*x*] is ∗-reversible under the induced involution defined by f*(*x*) = ∑_*i*=0_^*m*^ *a*_i_*x ^*i*^, for every polynomial *f*(*x*) = ∑_*i*=0_^*m*^ *a*_*i*_ *x* ^*i*^ ∈ *R*[*x*].

**Proof**

If part is trivial.

For only if, let *R* be Armendariz and is ∗-reversible, and let for some *g*(*x*) = ∑_*i*=0_^*m*^ *b*_*i*_ *x* ^*i*^ ∈ *R*[*x*], *fg* = 0. Then for any pair of coefficient *a*_*i*_ ∈ Coef(*f*) and *b*_*j*_ ∈ Coef(*g*), we have *a*_*i*_ *b*_*j*_ = 0, which implies that *b*_*j*_ *a*_i_* = 0 with *a*_i_* ∈ Coef(f*) and *b*_*j*_ ∈ Coef(*g*). Hence, we plainly get gf* = 0.

## 4. The Story of Some Minimalities

In [9] a ring *R* is defined to be right (respectively left) symmetric if for any triple *a*, *b*, *c* ∈ *R*, *abc* = 0, then *ac* *b* = 0 (respectively *b* *ac* = 0). If 1 ∈ *R*, then every right (or left) symmetric ring becomes symmetric, which returns the original definition of Lambek [6] of a symmetric ring.

Clearly, all commutative rings are left or right symmetric, symmetric, reversible, reflexive, duo (every right or left ideal is an ideal), semicommutative, and at least have a trivial involution. In this section, first we have obtained a criterion for rings to be noninvolutary and then we will find minimal (cardinality-wise) right and left symmetric, symmetric, reversible, reflexive, noninvolutary, and ∗-reversible noncommutative rings. 

*The following theorem is a criterion for rings to be noninvolutary.*

**Theorem**

A right (or left) symmetric ring which is not symmetric cannot adhere to an involution.

**Proof**

Let *R* be a right symmetric ring which is not symmetric. Assume on contradiction that *R* adheres to an involution ∗. If *abc* = 0 for some *a*, *b*, *c* ∈ *R*, then, because *R* is right symmetric, *ac* *b* = 0. Then (*ac* *b*)* = b*c*a* = 0 or b*a*c* = 0. Doubling the involution gives *cab* = 0 which means that *cba* = 0. Again, *abc* = 0 gives c*b*a* = c*a*b* = 0, and by the doubling of involution one gets *b* *ac* = 0 and so the right symmetry gives *bca* = 0. Hence, we conclude that *R* is symmetric, which is a clear contradiction. Similarly, one can prove that if *R* is left symmetric and it is not symmetric, then it cannot have an involution.

One can deduce from above the following:

**Corollary**

A right (or left) symmetric ring with an involution is symmetric.

**Example**

Consider the, so called, Klein-4 ring :*V* = {0, *a*, *b*, *c*} which is a Klein 4-group with respect to addition. The characteristic of this ring is 2 and the relations among its elements are

(30) $$\begin{array}{c} c=a+b,\;\;a^{2}=ab=a,\;\;b^{2}=ba=b \end{array}$$

(see [8, Example 1]. Erroneously it is considered symmetric there. This ring is not symmetric, simply because *abc* = *ac* *b* = 0 but *cab* = *c* ≠ 0. Similarly, *b* *ac* = *bca* = 0, but *cba* = *c* ≠ 0. Hence, this ring is right symmetric only. By Theorem 18, it is clear that *V* is not agreed to adhere to any involution.

The same is the case for *V* ^op^ which is left symmetric but not right symmetric and so is not symmetric. Hence, *V* ^op^ is also free from any involution.

Under these situations there is no question of ∗-reversibility on *V* and *V* ^op^.

Note that, up to isomorphism, the only noncommutative rings of order four are *V* and *V* ^op^. Hence, *V* and *V* ^op^ are the smallest (up to isomorphism) noncommutative right and left symmetric rings (as in Example 20), respectively. These rings are the smallest nonreduced (because *c* ≠ 0 is nilpotent), nonsymmetric, nonreversible, nonreflexive (*aRc* = 0, but *cRa* ≠ 0), nonabelian (*a* ^2^ = *a*, *ac* = 0, *ca* = *c*), nonduo ({0, *a*} is a right ideal of *V* which is not an ideal) and noninvolutary, so they are not ∗-reversible as well.

**Example**

The ring of strictly upper triangular matrices over *ℤ*_2_, namely, SUTM_3_[*ℤ*_2_] has only eight elements. It is noncommutative and is clearly symmetric, and, for the same reason, it is reflexive. But it is not reversible, because *e*_23_ *e*_12_ = 0 but *e*_12_ *e*_23_ = *e*_13_ ≠ 0. This ring is minimal with such properties. It adheres to an involution defined on its elements by

(31) $$\begin{array}{c} {}*:\left[\begin{array}{ccc} 0 & a & b\\ 0 & 0 & c\\ 0 & 0 & 0 \end{array}\right]\mapsto \left[\begin{array}{ccc} 0 & c & b\\ 0 & 0 & a\\ 0 & 0 & 0 \end{array}\right] \end{array}$$

but the ring is not ∗-reversible. In fact it is not ∗-reversible for any involution on it, because it is not reversible (see Proposition 5(2)).

The claim that it is minimal noncommutative symmetric and reflexive is clear, because all rings of order less than eight are commutative other than the two rings of order four, namely *V* and *V* ^op^, which we already have proved that are neither symmetric nor reversible. Hence, we conclude the following.

**Theorem**

A minimal noncommutative reflexive and symmetric ring is of order eight and is isomorphic to the ring of strictly upper triangular matrices over *ℤ*_2_, namely, SUTM_3_[*ℤ*_2_]. This ring is neither ∗-reversible nor reversible.

The same holds for the ring of strictly lower triangular matrices over *ℤ*_2_, namely, SLTM_3_[*ℤ*_2_].

The above rings are without one; for a ring with one we have a different minimal situation.

**Example 23**

[21, Example 2.5 and Theorem 2.6] states that if a ring *R* with identity is a minimal noncommutative symmetric ring, then *R* is of order 16 and is isomorphic to the ring *UM*_2_[*GF*(4)]; especially, *R* is a duo ring, where

(32) $$\begin{array}{c} UM_{2}\left[GF\left(4\right)\right]:=\left\{\left[\begin{array}{cc} a & b\\ 0 & a^{2} \end{array}\right]:a,b\in GF\left(4\right)\right\}. \end{array}$$

Moreover, in [23, Theorem 5] it states that if *R* (with identity) is a minimal noncommutative reflexive ring, then *R* is a ring of order 16 such that *R* is isomorphic to *UM*_2_[*GF*(4)] when *R* is abelian and to *M*_2_[*ℤ*_2_] when *R* is nonabelian.

We will prove that *UM*_2_[*GF*(4)] is a minimal ∗-reversible ring. For this we only prove that *UM*_2_[*GF*(4)] is ∗-reversible under some involution ∗. The rest follows from [21, Example 2.5 and Theorem 2.6], [23, Theorem 5] and Proposition 6.

Let us define an involution on the elements of *UM*_2_[*GF*(4)] by

(33) $$\begin{array}{c} {\left[\begin{array}{cc} a & b\\ 0 & a^{2} \end{array}\right]}^{*}=\left[\begin{array}{cc} a^{2} & b\\ 0 & a \end{array}\right]. \end{array}$$

Note that $\left[\begin{array}{cc} a^{2} & b\\ 0 & a \end{array}\right]\in UM_{2}[GF(4)]$ because *a* ^4^ = *a*, for all *a* ∈ *GF*(4).

This is an involution on *UM*_2_[*GF*(4)]. Indeed,

(34) [ab0a2]∗+[cd0c2]∗=[a+cb+d0(a+c)2]∗,([ab0a2][cd0c2])∗=[acad+bc20(ac)2]∗=[(ac)2ad+bc20ac]=[cb0c2]∗[ad0a2]∗.

We claim that this involution is ∗-reversible.

Note that *UM*_2_[*GF*(4)] is local and its only nontrivial ideal is its Jacobson radical

(35) $$\begin{array}{c} J\left(R\right)=\left\{\left[\begin{array}{cc} 0 & b\\ 0 & 0 \end{array}\right]:b\in GF\left(4\right)\right\}≅GF\left(4\right), \end{array}$$

so all other elements outside this maximal ideal are units. Thus, for any pair of non-zero elements *α*, *β* ∈ *UM*_2_[*GF*(4)], *αβ* = 0 if and only if *α* and *β* both belong to *J*(*R*); otherwise *αβ* ≠ 0. Hence, *J*(*R*) is a zero ring and so *αβ* = 0 implies that βα* = *βα* = 0. Hence, the claim is confirmed. This ring is obviously reversible as well. Hence, the following is proved.

**Theorem**

A minimal noncommutative ∗-reversible ring is of order sixteen and is isomorphic to the ring *UM*_2_[*GF*(4)].

**Example**

A ring is reversible but neither symmetric nor ∗-reversible.

Consider the group ring *ℤ*_2_(*Q*_8_): = {*x*_*t*_ : *t* ∈ *Q*_8_} where *Q*_8_ = {±1, ± *i*, ± *j*, ± *k*} is the group of quaternions. It is discussed in detail in [8, Example 7] that this group ring is reversible but not symmetric. A natural involution induced on *ℤ*_2_(*Q*_8_) is the involution on *Q*_8_ defined by ∗ : *g* ↦ *g* ^−1^, for all *g* ∈ *Q*_8_. In Example 4(4) it is verified that *ℤ*_2_(*Q*_8_) is not ∗-reversible. In fact, *ℤ*_2_(*Q*_8_) is not symmetric, so it cannot be ∗-reversible for any involution ∗ (by Proposition 6). The order of the ring *ℤ*_2_(*Q*_8_) is 256.

*A Comment on an Open Problem by Marks*. Marks in [8] posed a problem that whether there is any ring with the identity which has smaller size than *ℤ*_2_(*Q*_8_) and is reversible but not symmetric. We add here our comment that such a ring is not reversible with any involution as well (by Proposition 6).
